# Supramolecular association studied by Fluorescence correlation spectroscopy

**DOI:** 10.3389/fchem.2022.1042658

**Published:** 2022-10-31

**Authors:** Mercedes Novo, Wajih Al-Soufi

**Affiliations:** Single-molecule Research Unit, Biophysical Chemistry, Photophysics and Spectroscopy Group, Faculty of Sciences, University of Santiago de Compostela, Lugo, Spain

**Keywords:** Fluorescence Correlation Spectroscopy, supramolecular association, slow exchange, fast exchange, binding constant, association dynamics

## Abstract

A comprehensive description of a supramolecular system involves a full understanding of its thermodynamic and dynamic properties, as well as detailed knowledge of its structure. Fluorescence Correlation Spectroscopy (FCS) constitutes a powerful technique to acquire this information. Fluorescence correlation curves show a characteristic diffusion term that is related to the binding equilibrium constant or other thermodynamic properties of the supramolecular system. The association and dissociation rate constants of the binding process can be determined in FCS when the relaxation time of the binding is faster than the observation time—a regime called fast-exchange dynamics - in opposition to the slow-exchange regime. In all cases, structural information can be inferred from the diffusional properties of the supramolecular complexes. A short overview of the use of FCS for the study of supramolecular systems is given with examples which belong to the fast and slow regime.

## 1 Introduction

Supramolecular systems are assemblies of molecules maintained by non-covalent, weak interactions. They resemble biological systems regarding important aspects such as molecular recognition, self-assembly or host-guest binding. A key feature of these systems is their reversibility, which is a consequence of the weak intermolecular interactions between molecules and determines their function.

Structure, thermodynamics, and dynamics are needed to obtain a full picture of a supramolecular system (Bohne, 2011). Structure is defined by the stoichiometry of the building blocks and their spatial arrangement, whereas thermodynamics gives the stability and the specificity of the association. Dynamics is important to characterize the reactivity of these systems and understand their reversibility.

In contrast to molecules, supramolecular systems are highly dynamic with a high-order organization, which is often polydisperse and which determines their function (Bohne, 2014). Therefore, traditional chemical characterization methods in ensemble average are frequently insufficient and single-molecule approaches are needed to fully understand their behavior (Selvin and Ha, 2008). Single-molecule techniques have demonstrated to reveal individual behaviors typical of complex local environments and to give dynamic information of these systems while staying at the equilibrium, both in solution and in cell culture (Zander et al., 2002; Yanagida and Ishii, 2009). Among them, Fluorescence Correlation Spectroscopy (FCS) is a powerful technique that allows for the investigation of structure, thermodynamics and dynamics of supramolecular systems (Al-Soufi et al., 2005).

## 2 Supramolecular association studied by Fluorescence Correlation Spectroscopy

### 2.1 Fluorescence Correlation Spectroscopy

FCS is a free diffusion single-molecule technique monitoring the intensity fluctuations of a small number of fluorescent molecules (*N* = 1–10) as they diffuse through an open sample volume. The fluorescence probe has typically nanomolar concentration and the very small observation volume of femtoliters is defined by the focus of a collimated laser beam in a confocal microscope with a high aperture objective. A fluorescent molecule which diffuses into this sample volume is excited by the laser light and generates a burst of fluorescence photons which are collected and monitored as a function of time. Then the autocorrelation function of the intensity trace is calculated in order to extract the physicochemical information of the system under study (Elson, 2011; Wohland et al., 2020).

The observed fluctuations of the fluorescence intensity can be due to diffusion, chemical reactions, photophysical processes, structural changes or any other processes which modulate the fluorescence signal of the probe around the equilibrium value. Each of these processes appears in the autocorrelation function as a “term” at a typical correlation time. In all cases, the random diffusion of the probe through the solution leads to a term at the diffusion correlation time *τ*_*D*_, which is related to its translational diffusion coefficient *D* and hence to the size and shape of the diffusing particle. Therefore, the diffusion time yields direct information about the supramolecular systems, both regarding structure and thermodynamics of the species involved (Novo et al., 2011).

Typical processes in supramolecular systems such as host-guest interactions or self-assembly may also generate an additional correlation term, characterized by the reaction correlation time *τ*_*R*_, which is the relaxation time of the process. This reaction time yields dynamic information of the system at equilibrium with no need of an external synchronization.

In the following sections we will analyze these two terms in more detail, taking into account the type of dynamics of the supramolecular system.

### 2.2 Slow *versus* fast-exchange dynamics

The stability of supramolecular systems is characterized by the binding equilibrium constant *K* (note that often the reciprocal value, i.e. the dissociation constant *K*_*D*_, is used). A large value of *K* (or small *K*_*D*_) indicates high affinity between the building blocks. Thermodynamic studies also give information about the association stoichiometry and the number of binding sites, as well as the specificity of a certain ligand to different receptors.

However, no dynamic information can be inferred from the binding equilibrium constant (Bohne, 2014). For a certain value of *K*, a wide range of association and dissociation rate constants are possible (Novo et al., 2011). For example, for an intermediate 1:1 binding constant of 10^3^ M^−1^, the association rate constant *k*_*+*_ may vary within many orders of magnitude, with only a higher limit imposed by the diffusion controlled rate of about 10^10^ M^−1^ s^−1^. Given the relationship $K=k_{+}/k_{-}$ , it follows that the corresponding dissociation rate constant, *k*_*-*_, ranges from 10^7^ s^−1^ to values as low as 1 s^−1^ or even lower. Knowledge about these rate constants is crucial for the understanding of a supramolecular system, being *k*_*+*_ mainly related to the geometric and steric requirements for the binding and *k*_*-*_ to the strength of the interactions between the building blocks (Al-Soufi et al., 2005; Novo et al., 2007; Al-Soufi et al., 2008; Bordello et al., 2010; Bordello et al., 2012; Bordello et al., 2015).

The broad dynamic range of supramolecular association yields relaxation times as short as a few nanoseconds or as long as seconds. Diffusion correlation times are usually in the range from tenth to several thousands of microseconds, whereas the reaction correlation time can vary from a few microseconds to hundreds of microseconds. For a given experimental observation time in a FCS experiment two limiting time regimes have to be distinguished: “slow-exchange”, with long relaxation times with respect to the observation time, and “fast-exchange” where the binding dynamics is much faster than the observation time, with the reaction time at least one order of magnitude shorter than the diffusion time. Only in the second case dynamic information can be inferred from FCS, providing that the observed physical property changes during the binding process.

**FIGURE 1:**
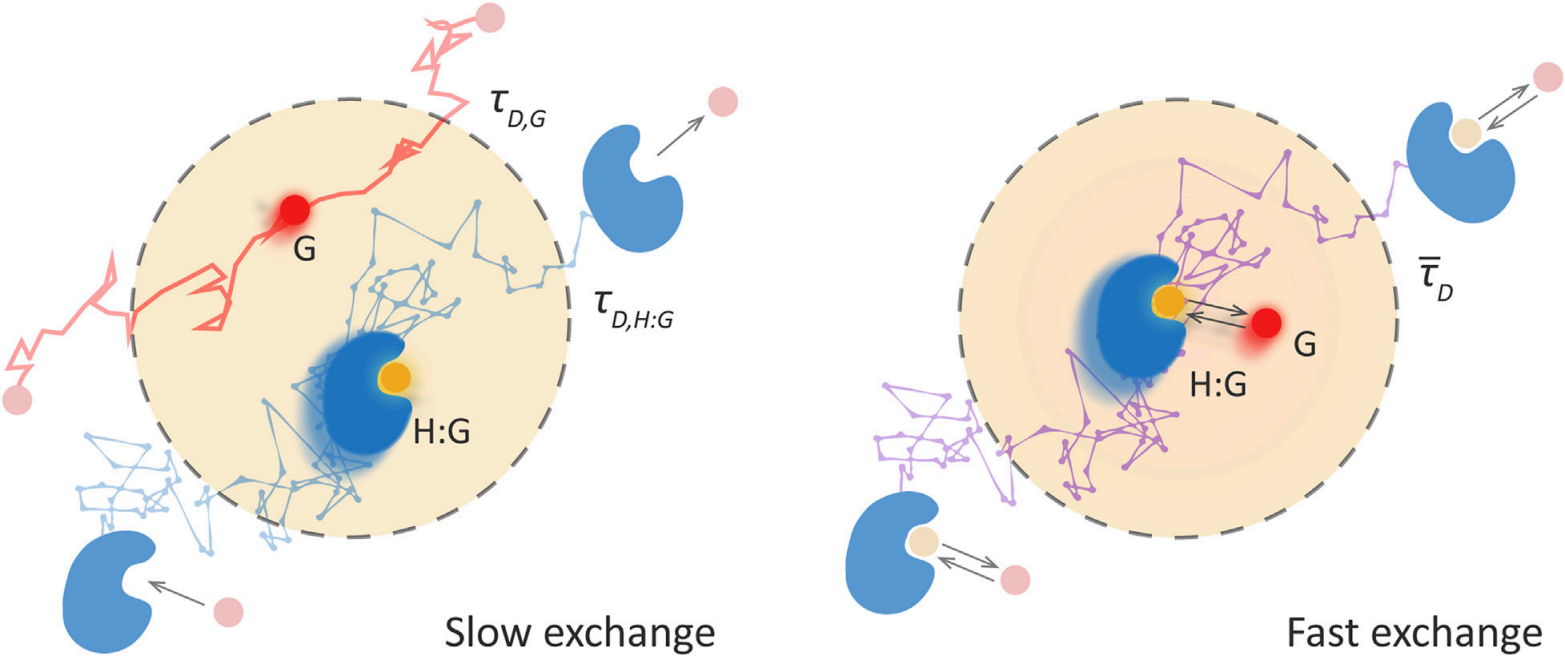
The two limiting regimes for the study of the association dynamics of host-guest supramolecular systems by FCS. The dashed grey line indicates the observation volume. Left panel: “Slow exchange”—the association dynamics are much slower than the diffusion time across the observation volume. The probability of an association or dissociation event during the observation is very low and the free guest, G, and the host-guest, H:G, are detected as separate species, each with its own diffusion time, τ_*D,G*_ and τ_*D,H:G*_, respectively. Right panel: “Fast exchange”—the association dynamics are fast enough to occur many times during the transit and, therefore, only one mean diffusion time, ${\bar{\tau }}_{D}$ , is observed. Its value depends on the fraction of time the fluorescent species is free or bound during the transit, which, on the other hand, depends on the equilibrium constant and the concentration of the host.

Figure 1 illustrates these two limiting regimes for host-guest supramolecular systems, slow-exchange dynamics on the left and fast-exchange on the right. The panel on the left, illustrates that the labelled guest passes through the observation volume either free or bound to the host, being the diffusion faster for the free guest. The rates of association and dissociation are too slow for these processes to occur during the time that the guest is observed. Therefore, two species are detected, the free guest and the host-guest complex, with different diffusional properties and contributions proportional to their equilibrium concentrations.

On the contrary, in the fast exchange regime (Figure 1, panel on the right), the labelled guest binds to hosts and separates several times during its diffusion through the observation volume. This leads to the detection of a single diffusing species, with a mean diffusion time which is a weighted average between that of free and bound guest. If, additionally, the fluorescence intensity of the labelled guest changes upon binding, also faster fluorescence fluctuations are observed that are directly related to the exchange dynamics.

In the following sections we will show the FCS curves typical of these limiting regimes and discuss the information that can be obtained from them. Then, different examples of supramolecular systems with these two dynamic regimes will be shown.

### 2.3 Slow-exchange dynamics

Slow-exchange dynamics is typical of highly stable supramolecular systems, with large values of the binding equilibrium constant and long relaxation times. In FCS experiments, this kind of systems show two diffusion correlation times, a shorter one related to the free labelled molecule and a longer one due to the bound labelled molecule, with a higher mass and slower diffusion. The contributions of these two correlation terms to the correlation curve depend on the concentrations of the two species and on their brightness (often the fluorescence properties of the dye change upon binding). No reaction term is observed in this case, since the relaxation time is longer than the diffusion time and no exchange takes place during the observation time.

**FIGURE 2:**
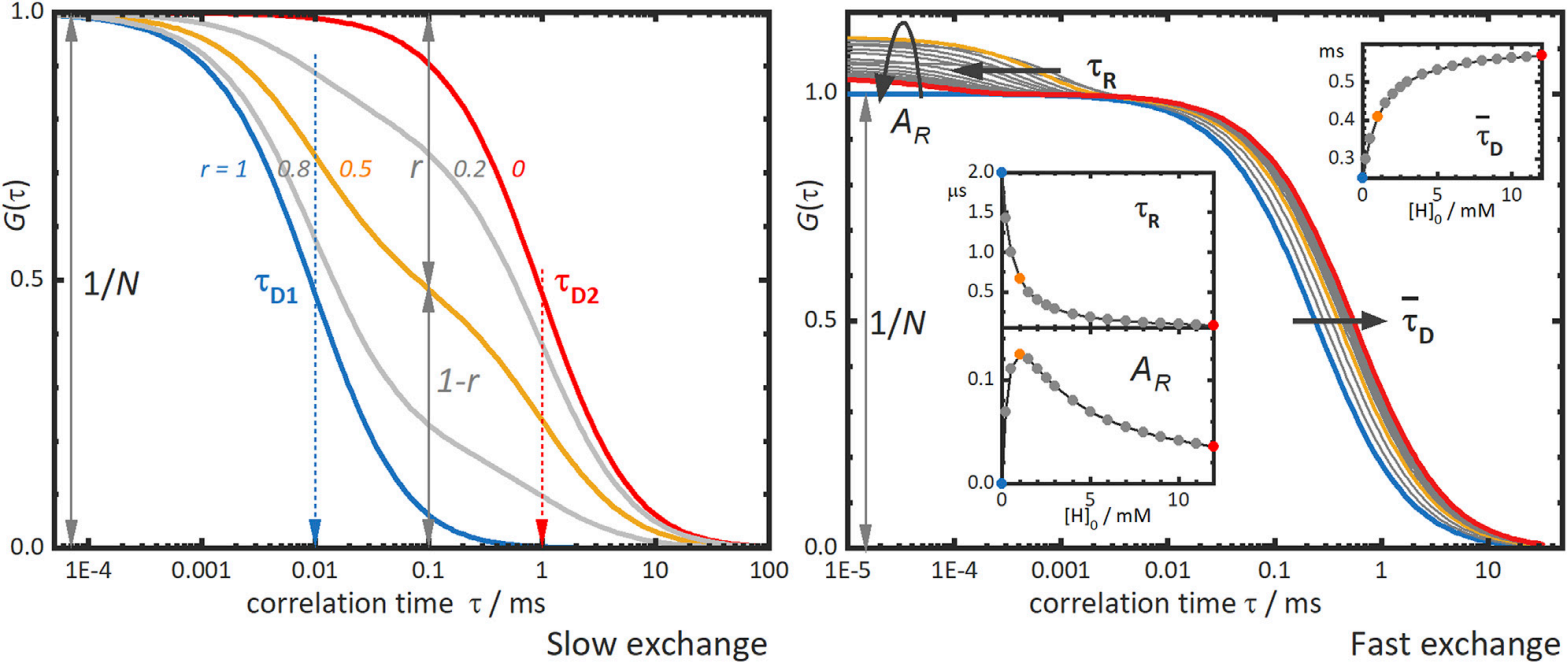
Simulated fluorescence correlation curves obtained in the slow and fast exchange regimes. Left panel: “Slow exchange”—the correlation curve shows two diffusion terms, one for each diffusing species, a fast one with τ_*D1*_, and another slower one with τ_*D2*_. In host-guest systems as that depicted in Figure 1, these species correspond to the guest with or τ_*D,G*_, and the complex with τ_*D,H:G*_, respectively. The different correlation curves in the panel correspond to different contributions of the two species to the curve as given by the parameter *r*. The blue curve with *r* = 1 and the red one with *r* = 0 would be obtained for solutions of only fast or slow species, respectively. The grey and orange intermediate curves correspond to mixtures of fast and slow species with *r*-values as indicated in the figure. Right panel: “Fast exchange”—the correlation curve shifts towards longer correlation times as the concentration of host is increased. The blue curve corresponds to that of free guest alone and the grey, orange, and red curves to increasing concentrations of host. The inserts show the dependence on the total host concentration, [H]_0_ *,* of the mean diffusion time, ${\bar{\tau }}_{D}$ , the relaxation time, τ_*R*_, and the amplitude, *A*_*R*_, of the reaction term. In both regimes, the amplitude of the diffusion term is given by the inverse of the total number of diffusing species, *N*.

Figure 2, left panel, shows typical FCS curves of supramolecular binding with slow-exchange dynamics. Two diffusion times are observed, a shorter correlation time *τ*_*D1*_ for the free labelled molecule and a second, longer time *τ*_*D2*_ for the supramolecular complex. The amplitude of the correlation curve is inversely proportional to the total number of diffusing species *N*. The contributions of each species to the correlation curve, giving by parameters *r* and *1-r* for *τ*_*D1*_ and *τ*_*D2*_, respectively, and depend on the concentrations and on the brightnesses of the corresponding species. The analysis of all these parameters yields thermodynamic information of the supramolecular system, such as the binding equilibrium constant and the stoichiometry of the supramolecular complex. However, small size differences between the building blocks of the supramolecular system may hinder the discrimination of high stoichiometry complexes, due to the limitation of FCS to differentiate two diffusion times which are close to each other.

Usually self-assembly systems present such slow-exchange dynamics, with intermolecular cooperative forces that lead to stable aggregates above a critical concentration of monomers. An example of this kind of systems is the early aggregation of the peptide Amyloid-β(1–42) (Aβ42), which is thought to initiate the neurodegeneration process in Alzheimer’s disease (Hardy et al., 2014). FCS titrations using a labelled peptide (Aβ42*) as probe were used to determine the concentrations and the diffusional properties of the species involved in the aggregation process (Novo et al., 2018). The results demonstrate that monomeric Aβ42 undergoes aggregation when its concentration exceeds a critical aggregation concentration (*cac*) of about 90 nM. Moreover, the aggregates formed are micelle-like oligomers, whose size and shape is independent of the incubation time or the peptide concentration.

### 2.4 Fast-exchange dynamics

As illustrated in Figure 1, right panel, in systems with fast binding dynamics, the association and dissociation processes take place during the time that the species diffuse through the observation volume. If, additionally, the fluorescent probe changes its brightness upon binding, both thermodynamic and kinetic information can be obtained in one and the same experiment.

The first effect of fast-exchange dynamics on the FCS curves is the observation of a single diffusion correlation term with a mean diffusion time, ${\bar{\tau }}_{D}$ , which is a weighted average of the diffusion times of the species forming the supramolecular system (Al-Soufi et al., 2005; Novo et al., 2011). The value of this mean diffusion time is related to the binding equilibrium constant and increases with the concentration of the supramolecular complex. As for the slow-exchange dynamics, the amplitude of the diffusion term is the inverse of the total number of diffusing particles, *N*. Figure 2, right panel, shows simulated FCS curves for a host-guest system with fast-exchange dynamics. The diffusion term moves towards longer times as the concentration of the unlabelled host is increased. From the variation of the mean diffusion time with the host concentration the binding equilibrium constant *K* can be obtained, as well as the diffusion coefficients of the free guest and the host-guest complex.

We showed how FCS can be used to determine high binding equilibrium constants in non-fluorescent host-guest systems (Granadero et al., 2010). A suitable labelling of the guest, usually the smaller molecule, allows for the determination of the binding constant from the change of the mean diffusion time with the host concentration. The very low concentration of the fluorophore which is needed in FCS makes it possible to have an excess of host even for systems with very high binding affinities.

Stoichiometry of the host-guest complexes can also be inferred from the profile of the variation of the mean diffusion time with the host concentration and the diffusional properties of the complex as compared to those of the free molecules. The sensibility of the FCS curves to different stoichiometries is especially high when a labelled guest is used and the size difference between guest and host is large.

The second effect in the FCS curves is the emergence of an additional term at shorter correlation times than the diffusion term (Figure 2, right panel). This “reaction” term is characterized by a reaction correlation time, $\tau_{R}$ , which is given by the relaxation time of the binding process, defined by the association and dissociation rate constants, *k*_*+*_ and *k*_*-*_, respectively, For a host-guest system, with a fluorescent-labelled guest, the reaction time is given by $\tau_{R}={\left(k_{+}\left[H\right]+k_{-}\right)}^{-1}$ , with [H] being the total concentration of the unlabeled host. The amplitude of the reaction term, *A*_*R*_, depends strongly on the brightness ratio between the free and the bound labelled molecule and shows a complex dependency with the binding equilibrium constant *K* (inset in the right panel of Figure 2). Both the reaction time and its amplitude vary with the concentration of host as shown in Figure 2, right panel, allowing for the determination of the different physicochemical parameters involved from FCS titrations.

Moreover, from the translational diffusion coefficients of the species determined form the FCS data, the limiting diffusion-controlled collisional rate constant of the association process can be estimated, making it possible to compare the observed association rate constant with this upper limit (Al-Soufi et al., 2005). This provides very useful information for the proposition of the corresponding kinetic mechanism of the binding process.

Our research group has pioneered the application of FCS to the study of fast supramolecular dynamics in host-guest systems (Al-Soufi et al., 2005). Fluorescent probes or fluorescent-labelled molecules suitable for FCS were used as guests to study different types of hosts, going from rigid cages such as cyclodextrins, which form inclusion complexes, to flexible micelles of surfactants and to biopolymers with binding capability.

Our FCS studies on the binding dynamics of pyronines with cyclodextrins explained the great differences in the stability of the formed inclusion complexes (Al-Soufi et al., 2005; Al-Soufi et al., 2008). The stability is dictated by the dissociation rate constants and not by the rate of the association process, as it may be expected. The association rate constants have the same order of magnitude for all systems under study, being among the highest reported for this kind of systems, but are significantly lower than the diffusion-controlled collision rate constant. From this information a two-step model is proposed. Firstly, an encounter complex is formed followed by an unimolecular inclusion reaction as the rate-limiting step. The rate of this second step seems to be controlled by geometrical and orientational requirements.

Comparing these binding dynamics of the relatively rigid cyclodextrins with those of the much more flexible micelles, one finds great differences in the association process. The association rate constants of dyes to micelles are much higher, and of the same order of magnitude as the estimated diffusion-controlled collision rate constants (Al-Soufi et al., 2008; Novo et al. 2011). The rate-limiting step is not the inclusion into the micelles, but the diffusion of dye and micelle to form the encounter complex. Based on this different behavior, we called micelles “soft cages”, with no geometrical or orientational requirements for the formation of the supramolecular complex, whereas cyclodextrins behave as “hard cages”, imposing geometric restrictions during the inclusion step.

We also used FCS to study the binding dynamics of small molecules to the minor groove of double stranded DNA (Bordello et al., 2012; Bordello et al., 2015). Again, the stabilities of the complexes formed were determined by the dissociation rate constants, quite high for the minor-groove binders under study due to the influence of the negatively charged fluorescent label. The association rate constants were at least two orders of magnitude smaller than the diffusion-controlled limit and were mainly responsible for the high specificity of the binders to AT sequences. The effect of salt concentration and the potential gliding mechanisms were analyzed with the help of a two-step mechanism with two types of encounter complexes (Bordello et al., 2012).

### 2.5 Structural studies of supramolecular systems by FCS

Beside thermodynamic and kinetic parameters, FCS studies yield also useful structural and geometric information. The diffusion correlation time *τ*_*D*_ is inversely proportional to the translational diffusion coefficient *D* and therefore related to the size and the shape of the diffusing species (Novo et al., 2011). Using the relationship between the diffusion coefficient and the mass, the size of the supramolecular complex can be obtained. This is especially useful for self-assembly systems, where the aggregation number of the aggregates can be estimated (Novo et al., 2018). From the dependency of the diffusion coefficient with the mass, information about the shape and conformation of the supramolecular complex or aggregate can be inferred (Al-Soufi et al., 2008; Bordello et al., 2009; Novo et al., 2018). Moreover, using the Stokes-Einstein relation the hydrodynamic radii of the supramolecular complexes can be estimated from the diffusion coefficients.

Our FCS study on the early aggregation of the Aβ42 allowed us to determine the conformational parameter *ν*, exponent of the diffusion coefficient *versus* molar mass dependency (*D* ∼ *M* ^–*ν*^), both for the monomeric species and for the aggregates (Novo et al., 2018). The value of *ν* = 0.44 for Aβ42 monomers indicates a random coil conformation with some structured regions of the peptide. For the oligomers a value of *ν* = 0.75 suggests an elongated conformation in agreement with the reported cylindrical geometry of the aggregates of a similar peptide (Yong et al., 2002). Furthermore, the size statistical distribution and the mean aggregation number of about 50 Aβ42 monomers per aggregate were obtained from the analysis of the experimental diffusion correlation times of the aggregates. From these parameters, also the hydrodynamic radii of the monomers and the oligomers could be estimated using Stokes-Einstein equation.

Similar studies of host-guest systems formed by a small fluorescent molecule and cyclodextrins yielded a power law for the diffusion coefficients of the complexes of *D* ∼ *M* ^−*0.4*^, a mass dependency in between the conformational parameter *ν* = 0.33 expected for compact spherical particles and the value *ν* = 0.5 which characterizes random coil conformation (Al-Soufi et al., 2005; Al-Soufi et al., 2008). This dependency was also found for micelles as hosts (Al-Soufi et al., 2008), allowing for the mean aggregation number determination.

## 3 Discussion

FCS is a very powerful technique for the study of supramolecular systems. Thermodynamic, dynamic, and structural information about these systems can be obtained from the same measurement or titration series. FCS is especially useful for the determination of association and dissociation rate constants in host-guest systems, where few other techniques are available. Nevertheless, to extract dynamic information from FCS measurements the system must fulfill two conditions: i) to be in the fast-exchange regime and ii) to show a significant change in the fluorescence intensity upon binding.

To comply with the first requirement the reaction time, *τ*_*R*_, must be much shorter than the diffusion time ${\bar{\tau }}_{D}$ , differing in at least one order of magnitude. Both, the reaction time, and the mean diffusion time, can be slightly tuned varying the host concentration (see Figure 2, right panel). More effectively, a significantly larger shift of the diffusion term can be achieved by changing the size of the observation volume, using different focus diameters. An increase of about one order of magnitude of the mean diffusion time can be obtained in this way. When, in spite of this, the reaction term and the diffusion term overlap, other single-molecule techniques with immobilized molecules may be used, such as smTIRF (Selvin and Ha, 2008). The second condition can be satisfied selecting a suitable fluorescent dye and the proper labelling site.
